# Mechanisms of ovarian aging in women: a review

**DOI:** 10.1186/s13048-023-01151-z

**Published:** 2023-04-06

**Authors:** Xiangfei Wang, Lingjuan Wang, Wenpei Xiang

**Affiliations:** 1 Tongji Medical College, Huazhong University of Science and Technology, Wuhan, 430030 China; 2 Department of Obstetrics and Gynecology, Tongji Hospital, Tongji Medical College, Huazhong University of Science and Technology, Wuhan, 430030 China; 3 Institute of Reproductive Health, Tongji Medical College, Huazhong University of Science and Technology, Wuhan, 430030 China

**Keywords:** Aging, Ovary, Ovarian follicle, Oocyte, Infertility, Genetics

## Abstract

Ovarian aging is a natural and physiological aging process characterized by loss of quantity and quality of oocyte or follicular pool. As it is generally accepted that women are born with a finite follicle pool that will go through constant decline without renewing, which, together with decreased oocyte quality, makes a severe situation for women who is of advanced age but desperate for a healthy baby. The aim of our review was to investigate mechanisms leading to ovarian aging by discussing both extra- and intra- ovarian factors and to identify genetic characteristics of ovarian aging. The mechanisms were identified as both extra-ovarian alternation of hypothalamic–pituitary-ovarian axis and intra-ovarian alternation of ovary itself, including telomere, mitochondria, oxidative stress, DNA damage, protein homeostasis, aneuploidy, apoptosis and autophagy. Moreover, here we reviewed related Genome-wide association studies (GWAS studies) from 2009 to 2021 and next generation sequencing (NGS) studies of primary ovarian insufficiency (POI) in order to describe genetic characteristics of ovarian aging. It is reasonable to wish more reliable anti-aging interventions for ovarian aging as the exploration of mechanisms and genetics being progressing.

**Supplementary Information:**

The online version contains supplementary material available at 10.1186/s13048-023-01151-z.

## Introduction

Ovarian aging is a natural and physiological aging process characterized by loss of quantity and quality of oocyte or follicular pool. Though aging is inevitable in human being, among the organs inside, ovary is believed to manifest characteristic of senescence in the relatively earlier stage of life span (about 35 years old), leading to age-related infertility. Moreover, as it is generally accepted that women are born with a finite follicle pool that will go through constant decline without renewing [1], which, together with decreased oocyte quality, makes a severe situation for women who is of advanced age but desperate for a healthy baby. The diminished fertility with advanced age has been described not only in historical populations but also in IVF (in-vitro fertilization) process [2–4]. Considering to a growing trend to delaying motherhood in contemporary society [5], which is partly owing to a higher level of income and education [6, 7], ovarian aging has become one of major obstacles for older mothers to bear healthy offspring. Hence, it is worthwhile to make intensive studies of biology, mechanisms and genetic characteristic underlying ovarian aging while broadening the horizon about original interventions to withstand it. Though there have been many excellent reviews related to ovarian aging recently [8–11], few of them systematically elucidated mechanisms both from extra- and intra-ovarian factors with an ignorance of the important interaction between the brain and the ovary. Moreover, what is not parallel with the well-developed sequencing technology is that there was a lack of comprehensive review demonstrating genetical mechanism involving not only Genome-wide association studies (GWAS studies) but also next generation sequencing (NGS) studies of primary ovarian insufficiency (POI) as well as a description of epigenetic characteristics. This article, by considering the shortages mentioned above, is meant to better the understanding of such a natural and physiological process, especially in human, and summarize novel findings and ideas as many as possible.

## Characteristics of ovarian aging in women

### Definition

Ovarian aging, also interpreted as female reproductive aging, is mainly defined by the loss of quantity and quality of oocyte or follicular pool [12]. The primordial follicles are developed during female fetal life and begin to decrease as soon as the oocyte pool is well established. After birth, the number of follicles keeps falling in the later life span, more exactly, from 1–2 million oocytes at birth to a few hundred in perimenopausal period [13, 14]. The progress of decline follows a program but not at a constant rate. Instead, the rate of decline increases with age, but whether the depletion rate gradually accelerates [15] or forms a sudden change at a given age was on debate [16]. In that case, by analyzing an independent data set, Knowlton et al. validated their previous hypothesis [15] and suggested a power model for the decline of ovarian nongrowing follicles [17]. During the whole reproductive life of female, with considerable follicles culminating to apoptosis, about only 500 oocytes succeed to ovulate eventually [18]. Though recent study about ovarian stem cell challenges the concept of unrenewed follicular pool, it is suggested to make only a minor difference in normal aging process due to its rarity [19]. However, as the success achieved by the use of female germline stem cells of human for oogenesis in vitro [20], these cells have the potential to be applied to fertility preservation in the future [21].

As the quantity of oocytes fall, the quality somehow declines as well. Oocyte quality, also regarded as developmental competence, is a manifestation of how well an oocyte can fulfill the whole process referring to completing meiosis, fertilization and sustaining embryonic development [22]. Though large part of the complicated mechanism associated with the decline of oocyte quality waits to be discovered, it seems that a few factors play a pivotal role in the regulating process, including mitochondria, spindle assembly, cohesion, telomeres as well as macro biomolecular damage and related increasing possibility of chromosomal nondisjunction which contributes to aneuploidy of oocytes as aging [23–25]. Nevertheless, some of their functions appear to be unclear or even controversy. And the mechanism will be discussed more detailly in the following section.

## Mechanisms of ovarian aging

**Fig. 1:**
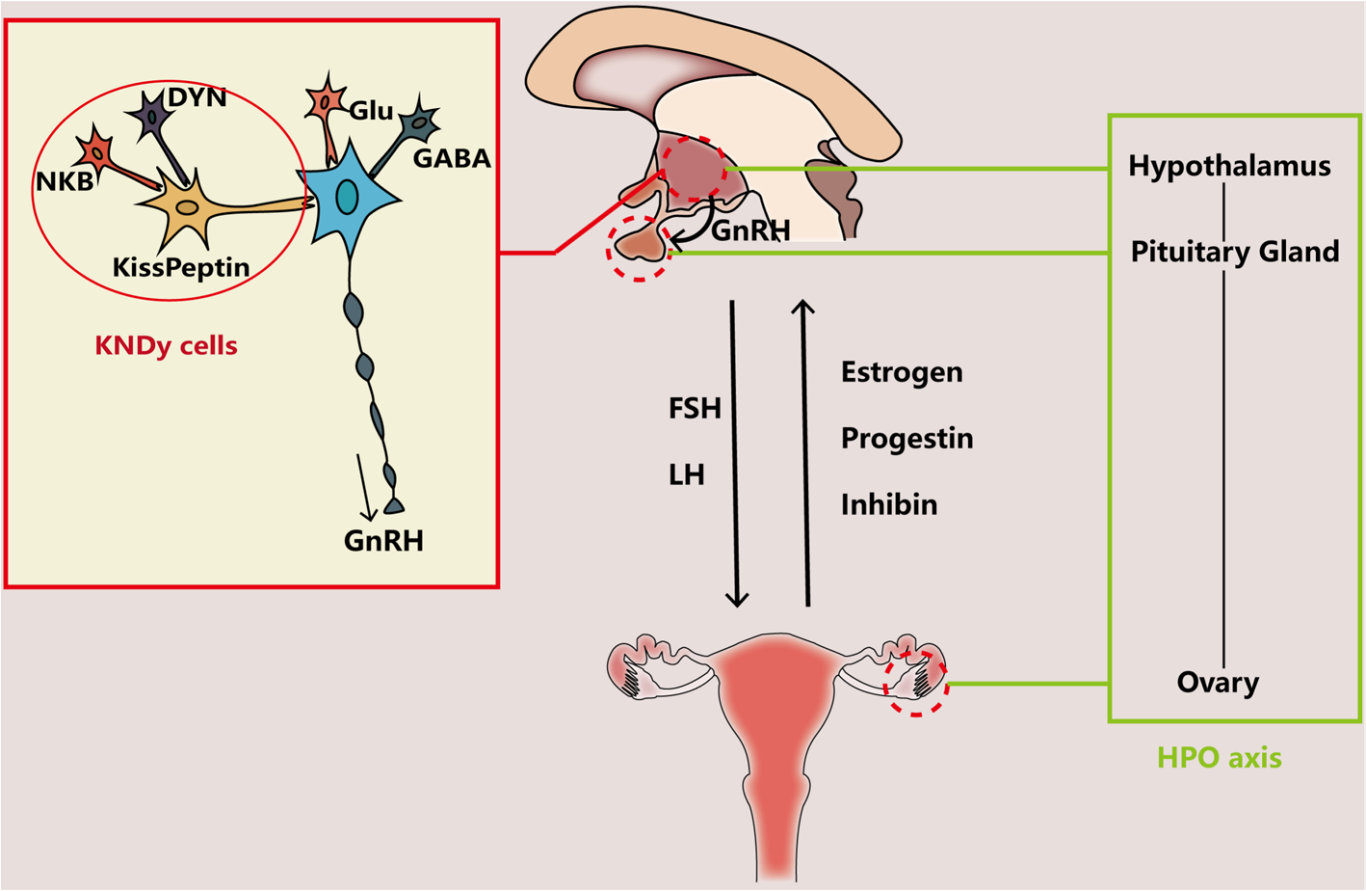
The interaction between brain and ovary through HPO axis by hormone release. Main neurotransmitter secretion in CNS that might affect the release of GnRH in hypothalamus include Glu, GABA and transmitters secreted by KNDy cells, including NKB, Kisspeptin and DYN. Brain interacts with ovary through hormones secreted from pituitary gland (FSH and LH) and from ovary (Estrogen, Progestin and inhibin). The relationship between ovary and brain can be described as HPO axis

The ovarian aging process is characterized by endocrine changes with unique hallmarks. Among those, the deficits of HPO (hypothalamic–pituitary–ovarian) axis play a decisive role in the onset of reproductive decline [26, 27]. Though myriad findings have proven that both the ovary and the HP (hypothalamic–pituitary) axis contribute to the reproductive aging process, it is still on debate which one is the trigger. Recent studies tend to ensure the leading role of HPO axis in reproductive aging, and here some related results are reviewed (see Fig. 1).

### The age-related dysfunction of hypothalamic–pituitary axis that leads to ovarian aging

So far, researchers have utilized rodents as a model to study the functional role of HPO in ovarian aging process, as a result of multiple changes in reproductively aging rodents parallel well with human. In 1964, endocrinologist Selmar Aschheim first carried out a study in which he transplanted the ovaries from young female rats with constant estrus cycle into old ones that lost the cyclicity and the latter failed to restore cyclic activity after the operation, revealing that the ovary is not the sole determinant factor of ovarian aging (reviewed in [28]). Similar research finished by Peng and Huang postulated an important role of HPA instead of ovary in reproductive senescence of female mice, since most of young mice whose hypothalamic were replaced by the old ones did not recover a normal estrus cycle and form corpora lutea whereas ovary transplantation succeed [29]. These early experiments hold the clue to the primary drive of ovarian aging and highlight the role of HPA during the reproductive aging process.

In line with rodent, evidence about neuroendocrine environment change exists in human, particularly the altered secretion pattern of GnRH and gonadotrophin. When treated with a single depot estradiol benzoate, the expecting LH surge occurs in seven of nine young women (ages 25–33), whereas only one of eight aged women responses an LH surge. The difference indicates a dysfunction of HPA in perimenopausal women [30]. Previous findings also document a gradual increase of gonadotrophin secretion that starts from the earliest reported age of 27–28 years and meets a dramatic increase after 40, with an earlier and more evident altered secretion pattern of FSH than LH [31]. As to LH secretion, more specifically, Matt et al. apply three types of independent and credible biomathematical models to test the LH concentration series and find an increase in LH pulse width as well as LH serge interval, while an attenuation in half-life of the hormone [32]. The changes of gonadotrophin secretion mentioned above occur before any visible menstruation or ovulation failure, and the altered secretion pattern of GnRH, which is produced by hypothalamus, might be partly responsible for the phenomenon [33, 34]. As a result, all of these evidences emphasize that the dysfunction of HPA is a contributing factor of ovarian aging in human.

Though the mechanism associated with the neuroendocrine change is still uncovered, some altered neurotransmitter secretion in central neural system that mainly take control of GnRH neurons might make an explanation partly. Glutamate, the ubiquitous neurotransmitter in CNS, has both ionotropic and metabotropic receptors expressed in the hypothalamus where the nuclei neural region that controls the secretion of GnRH, which is proved by immunocytochemical study. The colocation of VGluT2 (the glutamate transporter on vesicles of glutamatergic neurons) and GnRH neurons shows a coincidence of the two in the preoptic region and the median eminence where the cell body and the terminals of GnRH neuron locate in rodent’s brain respectively. More interestingly, restricted evidence leads to a possibility that the GnRH neuron might be able to release glutamate on its own in female rats [35, 36]. As aging, a decline of Glu level in aged female rates is conformed to the change of GnRH secretion that causes the postponed LH serge accompanied by lower amplitude [37]. At the same time, there is an attenuation of response to glutamate agonists in GnRH neurons, possibly due to a lost expression of NMDA1 mRNA (a type of glutamate ionic receptor) [38, 39]. In this case, both reduced Glu levels and decreased sensitivity of GnRH neurons to Glu might contribute to the alternation of GnRH secretion.

Another widespread amino acid neurotransmitter, GABA (Gamma-aminobutyric acid), also counts in the regulation of GnRH neural secretory activity. The GABA_A_ receptor with chloride to be the main charge carrier exists in the surface of GnRH neurons in rats [40]. And how GABA impacts on the secretion of GnRH can be explained by its association with glutamatergic neurons. In the indirect way, GABA inactivates the ionotropic glutamate receptor, leading to an attenuated rate of firing rate of GnRH neurons in mice, while when directly combining to the GABA_A_ receptor of GnRH neurons, it manipulates an opposite effect [41]. Study has also shown an amplified release of GABA in medial preoptic area when middle-aged female rats caught a reduced amplitude of LH surge with a postponed onset [42]. Still in the same study, researchers succeed in restoring normal LH surge in middle-aged rats by using GABA antagonist (bicuculline) and glutamate agonist (TPDC) to reach a regular Glu: GABA ratio, which supports the speculation that it is the balance of excitatory and inhibitory neurotransmitters that counts for a normal GnRH neural output [42, 43].

Followed by the examination of mutant gene in a consanguineous family with part of whose members suffer idiopathic hypogonadotropic hypogonadism, kisspeptin is believed to be another key macromolecular neurotransmitter regulating GnRH/LH secretion [44]. Similarly, study of human mutations also brought peptide Neurokinin B (NKB) into spotlight [45]. Together with peptide Dynorphin (DYN), the three of them form “KNDy (Kisspeptin/Neurokinin B/Dynorphin) hypothesis”, in which NKB activates neurons that release kisspeptin to stimulate GnRH neurons while DYN inhibits the kisspeptin neurons (for review, see Lehman et al. [46]). Not only is the arcuate kisspeptin neurons thought to be GnRH pulse generator in various species, which is detailed reviewed in the work of Herbison [47], but also KNDy cells occur to be impact factor of female reproductive aging process. For example, sufficient evidences have proven that the gene expression of KNDy has changed in postmenopausal women and female monkeys which might lead to an imbalance of excitatory and inhibitory input to GnRH neurons and cause neuroendocrine disorder during aging [48–51].

All in all, though not clear about the initial agent, the disorder of neurotransmitters in hypothalamus mediates the dysfunction of HPA, and ultimately might lead to ovarian aging. However, as mentioned above, both the initiating factor and the complicated interaction of various neurotransmitters are waiting to be figured out in further studies.

### The age-related alternation within ovary that leads to ovarian aging

**Fig. 2:**
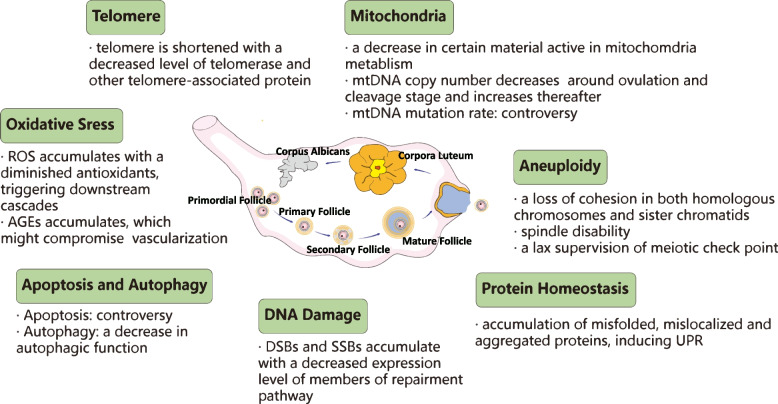
Overview of alternation in ovary with advanced age that contributes to ovarian aging

Besides for the dysfunction of HPO, the age-related changes within ovary itself, especially in oocyte might also attribute diminishing the quantity and quality of oocyte pool (see Fig. 2).

#### Telomere

Telomere is a complex of special repeated DNA sequence and shelterin proteins located at the end of eukaryotic chromosome. Its fundamental function is to protect the linear chromosome ends from being mistaken for a kind of broken ends and being wrongly repaired by cell itself or going through processes like DNA end-joining or DNA recombination [52]. However, as cell replicating, the telomere is inevitably depleted gradually and as soon as the telomere is shortened to a crisis point, the process of cell senescence can be launched. Due to its characteristic, the telomere can be regarded as a miotic clock in somatic cell [53]. Nevertheless, there are mechanisms to offset shortened telomere. Among those, the repairment done by telomerase is common within cells. The telomerase consisting of telomerase reverse transcriptase (TERT) and telomerase RNA component (TERC) is capable of replenishing the telomere through reverse transcription mechanism.

Unsurprisingly, besides for the senescence of somatic cell, there are numerous clues revealing the association between telomere and reproductive aging process. Characteristic of telomere in female ovary is distinctive due to the complicated folliculogenesis process as well as the inmate interaction between oocyte and granulosa cells along with cumulus cells. Using quantitative fluorescence in situ hybridization (Q-FISH) method, a study measures the telomerase length (TL) of human oocyte in different development stages and finds a decrease in TL from immature oocyte (11.41 ± 0.81 Kb) to mature MII oocyte (8.79 ± 0.86 Kb) which might be explained by accumulated unrepaired DNA damage in meiotic arrest [54]. In contrast, in cumulus cells which going through frequent miotic division, the TL is found to be longer in mature oocyte than immature ones using PCR measurement [55]. It is still unknown why TL of oocyte and cumulus cell manifest opposite trend in immature and mature oocyte, and future research that compare the telomerase activity between the two kinds of cells might provide some clues to this interesting phenomenon. Nevertheless, both oocyte quality and quantity are suggested to be associated with TL and telomerase activity. It has been proven that there is a positive relation of TL of cumulus cells and human embryo quality which suggests that the TL of cumulus cells can be another predictive biomarker for oocyte as well as embryo quality [55]. Moreover, the decrease in TL and telomerase activity of granulosa cells are also demonstrated to be associated with occult ovarian insufficiency in young women [56]. Besides for TL, the age-related change of components in telomerase (TERT and TERC) or other telomere-associated protein has been widely studied, and they show a compatible alternation with telomere shortening in mouse ovary [57]. As for further mechanism for TL shortening during reproductive aging process, the diminishing estrogen level might partly make an explain. In 2011, the study of Bayne et al. suggests the deficiency of estrogen which is an inevitable endometrium change in women undergoing menopause could attribute to the inhibition of telomerase and thus cause telomere shortening, leading to dysfunction of granulosa cell proliferation and ultimately contribute to defective ovaries [58]. This finding not only sheds the light on the mechanism of telomere shortening, but also connect the intra-change within ovary with extra-change in the endometrium environment and even more so, the HPO axis, which once again emphasize the significance of interaction between brain and ovary that leads to final ovarian aging.

#### Mitochondria

Functioning as the place for terminal catabolism of cellular energetic substrates in eukaryote, mitochondria is believed to be pivotal organelle that contributes to regular cell metabolism. Thus, not surprisingly, numerous clues have pointed out the relationship between the dysfunction of mitochondria and cell aging process. When referring to the ovary, there is no exception. To be more convincing, the successful oocyte rejuvenation obtained from cytoplasm transfer technology confirm, from another aspect, the link between oocyte quality and cytoplasm content, especially mitochondria and reveal the deterioration occurs in cytoplasm with advanced age (reviewed in [59]).

ATP level is largely decided by mitochondria function. In IVF process, within the normal-appearing MII stage human oocytes that are classified as “high grade”, there is a divergence observed in ATP content which might give rise to opposite outcome in the latter uterine transplantation and oocytes with ATP contents ≥ 2 pmol/oocyte were suggested to have higher embryogenesis and implantation potential [60]. Moreover, in mouse oocyte, the spindle disassembly, which is a conspicuous factor leads to aneuploidy and will be discussed later, can be caused by deterioration of mitochondria function and a decrease in mitochondria-produced ATP transported through mitochondrial permeability transition pores which is induced by oxidative stress (H_2_O_2_) [61]. Another chemical material active in the mitochondrial metabolism (including glycolysis, oxidative phosphorylation, and the tricarboxylic acid cycle), NAD+, is also supposed to be reduced in aging ovary. However, the introduction of nicotinamide riboside (a precursor of NAD+) can somehow reverse the age-related change of NAD+ level and bring fertility back to life [62].

When considering to mitochondria itself, overall, the dysfunction of mitochondria could also be roughly divided into quantity and quality variation. For the former one, it has long been studied how the abnormal quantity of mtDNA number along with mitochondria content is associated with ovarian aging process. Though the relationship between the number of mitochondria and mtDNA copy is not a linear one, on account of that a mitochondrion might contain several mtDNA copies, the mtDNA quantity can still roughly represent the mitochondria level and the ability to supply energy for the cell. In general, at the beginning of oogenesis, there is an obvious shrink of mtDNA copies (a couple hundred in mouse) in the primordial germ cells and afterwards a great amplification occurs, causing an increase in mtDNA copy number to more than thousand times to provide ample copies for further fertilization as well as implantation while these copies come from only a limited subpopulation of mtDNA molecules, which might pave the way for the transmission of mutated mtDNA to offspring and this will be discussed in latter paragraph. This conception is named of “bottleneck theory” [63, 64]. After ovulation, there is an almost quiescent period of mtDNA in early embryogenesis, and a boom in replication will not launch until the blastocyst stage [64]. And the age-related alteration associated with the number of mtDNA copy differs according to oogenesis stage. In the period around ovulation and cleavage stage, mtDNA copies are assumed to be more abundant in young female than aged ones, which has been proven by Konstantinidis et al. who use a microarray platform to test mtDNA quantity in polar bodies gained from human sample [65], and similar findings are also reported in bovine embryos [66]. As a matter of fact, as demonstrated by Fragouli et al., in embryos cultured just 2 days after cleavage stage, the trend of mtDNA quantity is upside down between young women and aged ones. The higher level of mtDNA also appears in the aneuploid blastocyst embryo, even independent of age [67]. The reason why there is a dramatic increase of mtDNA copies in blastocyst embryo from women with advanced age might be partly described by a compensatory mechanism, in which the abnormal amplification can be explained by an urgent need for enough ATP supply derives from embryonic stress and become a sign of developmental deficits [64]. As for the quality variation of mitochondria, the morphology has already prompted some clues. More specifically, an increase in numerical density (numbers of mitochondria per μm^2^ and per μm^3^ and also of the mitochondrial profile area (μm^2^)) in human oocyte has been discovered to be linked to progressive aging process, which reveals an age-related increase in mitochondria volume fraction [68]. Similar gain in mitochondrial volume is also frequently seen in mitochondrial myopathies since it might be a symbol of compensatory manifestation when compromising energy deficit. The quality of mtDNA which can be presented by mtDNA mutation rate is also said to be associated with ovarian aging and the ability to form a healthy embryo [67, 69, 70]. Recently in 2020, Yang et al. first quantified mtDNA mutations in human oocyte using next-generation sequencing (NGS), proving that mutations accumulates with aging [70]. However, this result is conflicting with previous report, in which Boucret et al. found oocyte to be not prone to accumulate mtDNA heteroplasmic mutations during ovarian aging [71]. The difference may be due to the use of diverse sequencing method. Specifically, the application of Ion Torrent Proton chip sequencing by Boucret et al. might miss mutations with frequency lower than 2%, which is too abundant to ignore in elder women as found by Yang et al. This reminds researchers that the quantification of mtDNA mutations and copy numbers is closely related to the detection method. As the development of sequencing method, more precise relationship between mitochondria and ovarian aging will be uncovered.

#### Oxidative stress caused by ROS and AGEs

Reactive oxygen species (ROS) has long been concerned as an inducement of aging process in organs by establishing oxidative stress and giving rise to the damage of biomolecules including proteins, DNA and lipids, and a related fascinating mechanistic theory named the Oxidative Stress Theory is established as a fundamental theory that explains aging process [72, 73]. In the ovary, however, as a normal byproduct during metabolism, ROS, mainly referring to superoxide (O2•-), hydrogen peroxide (H_2_O_2_) and hydroxyl (OH•), are indispensable material participating in physiological activities of ovary when at balanced level. The ovulation process, as an example, resembles to acute inflammation response for the LH surge launches numerous gene expression with plenty of them related to inflammation [74]. And it has been suggested that ROS plays a significant role during such an inflammation-like response, by inhibiting Epidermal Growth Factor Receptor (EGFR) phosphatases or Mitogen-activated Protein Kinase (MAPK) phosphatases to activate EGFR and generates a signaling cascade [75]. Nevertheless, other physiological activities are also said to be associated with ROS including follicular development and atresia, corpus luteum regression and apoptosis [76]. Moreover, physiologically, oxidative defense made of enzymatic and non-enzymatic antioxidants functions as the scavenger of ROS in case of damage caused by it. But as soon as the balance of oxidative stress and oxidative defense is broken, side effect of ROS will overpower its physiological function. Within ovary, since the primary oocyte that rest in the first meiotic prophase but is still active in metabolism might last for several years before ovulation, which makes the oocytes severe vulnerable to the chronic oxidative stress and the pathological effect induced by ROS mainly manifests in the changes of cellular biomolecules [77, 78]. A study applying 4-hydroxynonenal, nitro tyrosine, and 8-hydroxy-20-deoxyguanosine as markers for lipid, protein, and DNA damage, respectively, has proven the existence of age-related accumulated impairment of biomolecules caused by ROS, at least in mouse ovary [79]. A damaged biomolecule system including oxidized proteins, changed membrane lipid composition as well as impaired DNA,all together, triggers a series downstream cascades and ultimately impact on oocyte meiosis, follicular atresia, GC apoptosis as well as other cell activities, moreover, together with additional affected mechanism associated with mitochondria injury, telomere shortening and apoptosis (reviewed by [80–82]). Besides for natural ovarian aging process, the oocytes might encounter another circumstance with more sever threat caused by ROS, which is ubiquitous in IVF (in vitro fertilization), where the oocyte has to experience a set of operation in a hyperoxia environment (with a 5% CO2 and 20% O2 atmosphere at assisted reproduction laboratories) before returning to human uterus (with a 2%-8% oxygen tension) [83]. Cultivated in such a hyperoxia environment might induce oxygen toxicity that is able to drive cytoplasmic aberrations including aggregation of cytoskeleton components and endoplasmic reticulum condensates, which should be avoid to the greatest extent to maintain embryo quality [84].

Another material responsible for the oxidative stress in ovary is advanced glycation end‐products (AGEs). Formed by reactive carbonyl species and free amino groups residing, the AGEs are stable products gained from a series of nonenzymatic reactions called the Maillard reaction [85, 86]. A common in vivo component of AGEs is carboxymethyl lysine, and besides, the intake of food prepared at high temperature could also be an important source of AGEs [87, 88]. AGEs mainly function as a ligand to the corresponding transmembrane receptor (RAGE) and activate the transcription factor nuclear factor (NF-κB), resulting in proinflammatory gene expression and the generation of ROS [89]. Studies have proven that AGEs accumulate within the ovary of women as aging [90, 91]. First reported by Diamanti-Kandarakis et al., given to the immunohistochemical results, they identified the existence of AGEs, RAGE and activated NF-κB in both normal and polycystic ovary in women [91]. AGEs are suggested to take part in ovarian aging process due to its particular interaction with ROS and the characteristic to introducing oxidative damage to the ovarian microenvironment [92, 93]. Moreover, there is a negative correlation of a soluble isoform of RAGE and age in human oocyte plasma, as well as a positive correlation with vascular endothelial growth factor (VEGF) and age in follicular fluid [94], indicating that AGE-RAGE-VEGF signaling may make an influence in the process of ovarian aging. Considering to that elevated VEGF in follicular fluid was associated with adverse outcomes of IVF [95], it is reasonable to hypothesis that the AGE-RAGE interactions may stimulate VEGF production, leading to reproductive dysfunction. Last but not least, the accumulation of AGEs in human follicular fluid is able to impair oocyte quality, probably due to inducing the production of proinflammatory cytokines IL-6 and IL-8 and changing the follicular microenvironment according to experiment on human granulosa-lutein cells [96].

#### DNA damage

Since the primordial follicles in women’s ovary remain in the first meiotic prophase until they are recruited, the time interval will make it possible for oocytes to suffer from various hazard that might introduce damage to DNA and other biomacromolecules. ROS, as an example mentioned above, is an endogenous ubiquitous harmful factor [97]. Besides, exogenous stressors including ionizing radiation and chemotherapy drugs, which can be frequently seen during oncotherapy, as well as other toxicants from environment are also believed to be capable of attacking DNA [98]. The lesion of DNA often emerges in forms as single-strand breaks (SSBs) and double-strand breaks (DBSs). The later one could arise by two SSBs in close proximity or by one SSB formed at the location of DNA-replication apparatus and is suggested to be the most serious DNA damage type which is also difficult to repair [99]. There are two main DNA repairment pathways ensuring the integrity of DNA. First is the homologous recombination (HR) pathway, in which one or two initial strands with DBSs will invade another DNA single strand with homologous sequence, and repair itself according to homologous DNA strand to form crossover or no-crossover products. The initiation of HR involves the detection of DSBs by MRN complex (composed by double-strand break repair protein MRE11, DNA repair protein RAD50 and Nibrin (NBN)). Thereafter, the complex recruits and activates the serine-protein kinase ATM (ATM) and then MRE11 to process the broken ends to form single stranded DNA. After DNA repair protein RAD51 (RAD51) binds to the ssDNA to assemble the presynaptic filament, it will capture a duplex DNA molecule and begin to search for homology. Then it comes to the actions of polymerases, nucleases, helicases and other components to achieve DNA ligation and substrate resolution [100]. As to another repair pathway, the nonhomologous DNA end joining pathway, it can repair DSBs by forming direct ligation between the two ends after the clearance of any single stranded DNA overhangs. In somatic cells, the error-prone NHEJ pathway is able to function in any phase of cell cycle, while HR generally operate in S to G2 phase where sister-chromatid template is available [98]. In a word, the two pathways cooperate and competes with each other to ensure an effective DNA repair system.

In the oocyte, it appears that there are different repairment patterns according to distinctive oocyte developmental stage. In the early stage, the primordial follicle is sensitive to DNA damage, and will arrest at cell cycle or go through apoptosis as long as the accumulated DNA damage is out of tolerance and not able to be repaired [101]. The protein ATM plays a central role in the surveillance system of DNA damage. Since the primordial oocyte arrest at G2/M, HR pathway is suggested to be the main repair pathway, and after the MRN complex detect and bind DSBs, one component of the complex, the NBN protein, is allowed to interact with ATM dimers which contributes to autophosphorylation of ATM [102]. Then follows the phosphorylation of a histone protein, H2AX, to generate γH2AX, resulting a downstream pathways of DNA repair or cell cycle arrest [103]. There are abundant evidence demonstrating that DNA damage is associated with a diminished primordial follicular pool. In 2013, Titus et al. found a sharp decline of the expression of *BRCA1, ATM, RAD51, MRE11* in women after 36 years old, indicating that DNA repair system is impaired as aging [104], which is also true in aged rats whose expression of DNA repair genes, *Brca1, Rad51, Atm, Ercc2, and H2ax* are found to be decreased in primordial follicles both in real-time PCR and western blot studies [105]. The sharper decline in DNA repair ability of oocyte leaves oocyte reserves more prone to environmental toxicants ether endogenous or exogenous and thus, this situation prompts the accumulation of DNA damage giving to overwhelming stress to the oocyte and finally, push it to go through apoptosis or cell cycle arrested. As for the oocyte quality, the compromised ATM-mediated DNA DSB repair pathway is also responsible for increasing possibility of aneuploidy as aging because it induces the following decline of BRCA1 function in meiotic spindle assembly or reduced chiasmata (points of DNA cross overs) frequency [104, 106]. About the gene *Brca1*, it is not surprising that the *Brca1* deficiency heterozygote mice illustrates higher percentages of follicles with DSBs in postnatal life and even with lower ovarian reserve [104]. There are similar results in *BRCA1*-mutated women, who demonstrates a low response to ovarian stimulation which might indicate the relationship between DNA repair and ovarian function [107]. And for cases in the monkey, immunofluorescence results directly demonstrates a decline of the expression of *BRCA1* with advancing age in both oocytes and granulosa cells of primordial, primary, and secondary follicles [108]. Except for the sever DSB damage, other type of DNA impairment is also suggested to be related to ovarian aging, including lesions emerging on a single strand of DNA such as SSBs or apurinic/apyrimidinic sites, which can be repaired via base-excision-repair process, while in ovary of mice, the deficiency of key base-excision-repair enzyme, DNA polymerase β, will lead to ovarian aging [109, 110]. However, as the primordial follicles grow up to mature oocyte, DNA damage response somehow becomes limited. Though without abundant studies and evidence, it has been hinted that oocytes of mice are able to undergo germinal vesicle breakdown and enter M phase with low levels of DNA damage, indicating that the surveillance ability of G2 check point is diminished in oocyte which might be caused by the low sensitivity of ATM-dependent checkpoint [111]. In this case, M phase checkpoint can be a suitable substitute for the low efficiency G2 checkpoint in oocyte to monitor DNA integrity of mature oocyte, which is invoked and modulated by the spindle assembly checkpoint (SAC). Since the function of SAC is impaired in aged mice, the increasing possibility of abnormal embryo can be partly explained [112]. Still, a lot of work needs to be done to figure out how and why the DNA damage response in the ovary alters as aging with other novel DNA repairment pathways rolling in. Las but not least, as to the DNA damage, interestingly, studies in long-living Ames dwarf mice elucidate a negative association between growth hormone secretion and oocyte DNA integrity [113, 114], which again, supports that the cooperation of HPO axis and ovary itself maintain the reproductive health of ovary.

#### Protein homeostasis

As another indispensable biomacromolecule within the body, homeostasis of protein can also be a trigger to aging process when impaired. As a matter of fact, there is a network in the cell that keeps proteins in the correct concentration, conformation, subcellular location and in harmonious as well as stable cooperation with each other, which is officially named as protein homeostasis (proteostasis). However, as aging, the accumulation of misfolded, mis-localized and aggregated proteins will break the balance made by proteostasis and the collapse of proteostasis might lead to cell death which macroscopically contributes to advancing aging process or even pathological body condition [115]. To maintain the proteostasis and respond to stress, cells of organism have evolved an elaborate and conserved system with several organelle-specific stress responses being well studied, including the heat shock response in cytosol, and the unfolded protein responses (UPR) in endoplasmic reticulum as well as mitochondria [116]. When it comes to the ovary, similar research results have been elucidated. Lately in 2017, Wang et al. have found an association between oocyte quality and mitochondrial UPR in mice [117]. In the study, they targeted at the gene *Clpp*, which is in charge of expression of caseinolytic peptidase P (CLPP) in mitochondria matrix that works at cleaving misfolded proteins before they are exported to cytosol and go through downstream pathway [118]. After assessing the ovarian reserve and oocyte quality of *Clpp*^*−/−*^ mice, they found a decrease in the number of MII oocytes compared with wild type and that whether matured in vivo or in vitro, there is chromosome misalignment observed at the first and second meiotic metaphases in oocyte, which all around, illustrates damage brought by the deletion of *Clpp* in both oogenesis and oocyte quality and makes the *Clpp*^*−/−*^ mice resemble to an ovarian aging phenotype [117]. Moreover, when performing RNAseq analysis in GV stage oocytes of 3 and 6 months *Clpp*^*−/−*^ mice, the mTOR (mammalian target of rapamycin) signaling, which can preserve primordial follicle pool when inhibited, are suggested to be activated, probably accelerating oocyte depletion [117]. Another research using *Clpp*^*−/−*^ mice reports similar results, in which it is hypothesized that impaired surveillance system of protein damages mitochondria function and dynamics both in cumulus cells and in oocyte and contributes to ovarian aging phenotype [119]. Interestingly, when the deletion of *Clpp* is limited to granulosa/cumulus cells, the oocyte quality and number seems to be normal rather than previously reported when deleting *Clpp* in all cells, indicating that the alteration in oocytes must be induced from cells other than granulosa cells, which is likely from oocyte itself [120]. There are also a few research that study the association between endoplasmic reticulum stress and reproductive physiology as well as pathology (reviewed by [121]). However, because of a lack of correlation with ovarian aging process, these studies would not be discussed in depth here. Nevertheless, when referring to the factors that may do damage to proteostasis, oxidative stress that mentioned in the above section is always suggested to be remarkable. Recently, 4-hydroxynonenal, which is a product generated by oxidative stress induced lipid peroxidation and can be added to protein to deteriorate proteosatsis, has been found to be more defective in aged oocyte considering to an elevated vulnerability [122]. Though it is known that cells are capable of removing protein with incorrect decoration, conformation or subcellular location by the aid of stress response system that can regulate proteome, it is the attenuated proteome activity that increases the vulnerability of aged oocyte to damaged proteins such as 4-hydroxynonenal–adducted-α-tubulin, stops aged oocyte from maintaining homeostasis of proteins and contributes to impaired oocyte quality [123]. In summary, the proteostasis within cells of ovary is facing challenges mainly brought by oxidative stress all the time, but attenuation of stress respond ability as aging might open a gate to the accumulation of damaged protein which leads to the collapse of proteostasis and finally drive cells into apoptosis or autophagy.

#### Apoptosis and autophagy

Named by Kerr et al., the term apoptosis is a morphological description for a certain type of programmed cell death with morphology characteristic such as rounding-up of the cell, chromatin condensation, nucleus fragmentation, disappearance of ultrastructural modification of cytoplasmic organelles, plasma membrane blebbing, yet together with the maintenance of an intact plasma membrane until late stages of the process [124, 125]. Within the ovary, almost 99% of follicles end in atresia, most via apoptosis [126]. The survival of an oocyte is closely related to granulosa cells that revolve around. It is the growth factors, nutrients and survival factors provided by the granulosa cells that largely influence the solution to the live-or-die problem [127, 128]. Moreover, granulosa cells are capable of keeping the oocyte from the threat brought by ROS, which is a significant inducer of oocyte apoptosis [128]. Specifically, oocyte go through apoptosis mainly via mitochondria or BCL-2 (apoptosis regulator) mediated pathway and surface death receptor mediated pathway, with a number of players involving in the process [128–131]. However, though there is an undeniable role played by apoptosis of oocyte and granulosa cells during female reproductive life, the relationship between apoptosis and ovarian aging still remains unclear with existing controversial research results [132]. Early in 1997, Nakahara et al. has suggested a positive correlation between the incidence of apoptotic body in mural granulosa cells and poor IVF outcome [133]. Partial different from them, Lee et al. draw the conclusion with more attention to the apoptosis in cumulus cells, who, however, still claim an association between apoptosis of cumulus cells and oocyte quality, though with a limited sample size of 4 patients in old group [134]. Nevertheless, it should be noted that early studies were often based on light microscopy and assessed pyknotic cell count or index to quantify apoptosis. With a development in flow cytometry which is capable of examining granulosa cells from a single follicle with a known size and exclude the influence of white blood cells, contradictory research results occur. Regan te.al, for instance, after individually examining granulosa cells collected from 198 follicles by flow cytometry, find reduced apoptosis in granulosa cells of old women at the time of dominant oocyte selection and in the largest follicles compared to younger female. This alternation might be ascribed to a reduced expression of Bone morphogenetic protein receptor type-1B (BMPR1B) along with FSH and LH receptors [135]. After adjusting experimental error of methodologies, it seems that apoptosis is no longer assumed to be positive related to oocyte quality, nor a suitable predictor of clinical results. Rather, apoptosis of granulosa cells might be more important as a component of normal follicular development reflecting the mitogenic growth of the follicle and changes in accordance with developmental stage [132].

As research progress in ovarian aging, another type of programmed cell death, the autophagy, now has attracted researchers’ attention more than ever before. Similar to apoptosis, autophagy also refers to a typical morphological alternation of cell death, which is characterized by massive autophagic vacuolization of the cytoplasm [125]. Three different types of autophagy have been well studied, including macro autophagy, micro autophagy and chaperone-mediated autophagy, in which macro autophagy is assumed to be the most classic involving the formation of double-membrane vesicles (autophagosomes) loaded with selective cargo and delivering to lysosomes through vesicular fusion [115, 136]. In organisms, autophagy always functions as a double-edged sword especially in disease condition, as it exhibits adaptive response when facing with stress due to its ability to dump the waste, while might also lead to cell death or morbidity at the same time [137]. And there is no doubt that autophagy plays a significant role in the whole process of oocyte development [137, 138]. In aged rats’ ovary, the expression levels of gene *Atg5*, *Atg12*, *Atg16L*, *Beclin1* and *Lc3B*, which are closely related to the initiation and maintenance of autophagy, significantly decreased, suggesting a correlation between ovarian aging in these rats and a decrease in autophagic function [139, 140]. And the treatment of rapamycin, which is a mammalian target of mTOR inhibitor and is able to activate autophagy pathways, is proved to promote the nuclear and cytoplasmic maturation of young and aged porcine oocytes, contributing to improvement of oocyte quality, which exaggerates the significance of autophagy in ovarian aging process from another aspect [141]. As to how the autophagy affects ovarian aging process, it seems that ROS might somehow works as a bridge, for example, by generating lysosomal lipofuscin, which is composed of cross-linked protein residues and accumulates as aging due to its nondegradable characteristic, causing an attendant reduction in the efficacy of lysosomal degradation to attenuate oocyte quality [138, 142]. And a review has suggested a link between mitophagy, which refers to autophagy of mitochondria, and oocyte quality, in which Shen et al. summarize that mitophagy is responsible to remove aberrant mitochondria in the stage of oogenesis rather than after the stage of oocyte formation, giving to possible transmission of damaged mitochondria to offspring [143]. Though both apoptosis and autophagy account for the cell loss in ovary, as studies move on, the different roles played by apoptosis and autophagy in ovarian aging has been reexamined and still, no definite conclusion has been made, further studies are despairingly in need [144, 145].

#### Aneuploidy

Frequently recognized in the previous section, aneuploidy, referring to abnormal number of chromosomes contained in oocyte, whether more or less, largely determines oocyte quality as the fertilization of an oocyte with aneuploidy always causes disability in embryos which often leads to implantation failure or miscarriage in early embryo developmental stage, and even if the embryo strives to live to birth, some physical, behavioral, intellectual or developmental impairment will follow during the whole life [146, 147]. The direct factor leading to aneuploidy is about the wrong chromatin segregation which can be seen in both meiosis I and II [148, 149]. And on account of the increasing possibility of aneuploidy in oocyte or embryo with advanced age (> 35), more specifically, a J-shape demonstrated in maternal age curve for incidence of aneuploidy [150], it is reasonable to believe that errors of chromatin segregation that take place in meiosis process are indispensable part of ovarian aging mechanism. In mammals, at least two mechanisms of aneuploidy related to aging process are well studied, which collectively refers to a loss of cohesion in whether homologous chromosomes or sister chromatids, and a disability of spindle is also involved [149, 151]. During meiosis process, a consecutive divisions occur first in homologous chromatins (MI) and thereafter, in sister chromatids (MII), though in oocyte the successive process might last for years. Between homologous chromatins, concerning to a necessary step of homologous recombination, parental and maternal chromatins will be linked together by synaptonemal complex to form a configuration called bivalent and crossovers emerge to achieve genetic recombination. After a long time period of arrest in metaphase of meiosis I, the oocyte stimulated by a surge of LH proceeds meiotic process by breaking down nucleus and assembling a meiotic spindle [149]. However, spindle assembly in human oocyte is characterized by chromatin-dependent microtubule nucleation instead of centrosome-dependent. Spindle disability contributed by transformation between multipolar spindle intermediates and a bipolar spindle, which might lead to an opposite orientation of sister kinetochores when attachment of sister kinetochores and spindle poles happens [152–154]. Moreover, the wrongly attachment of kinetochores are supposed to be under surveillance of SAC. However, the gatekeeper somehow often neglects the errors and fail to impede cell division in oocyte [155], which, together with spindle disability, develops a relatively high baseline level of errors in chromatin segregation. Nevertheless, even though attachment takes place correctly, it is alarming that there is a split nature of sister kinetochores in human oocytes, driving sister chromatids to be segregated much earlier than they are supposed to be, and what is more worrying is that such a situation deteriorates as aging in human [156, 157]. In old mouse, the reason why cohesion between sister chromatids diminishes, demonstrating by increased distance between sister kinetochores, can be partly explained by a loss of chromosome-related meiotic recombination protein REC8 homolog (REC8) whose responsibility is to protect chromatins and sister chromatids from segregation [158]. And since the total REC8 level has been estimated to be of no difference between young and old oocyte, it is possible that loss of cohesion with advanced age is not able to be effectively replaced, which has been proven in other study [158, 159]. Nevertheless, similar alteration has illustrated to occur in meiotic cohesion protein, structural maintenance of chromosomes protein 1B (SMC1B) as well as cohesion subunit SA-3 (STAG3) that is located between sister chromatids [160]. In human oocyte, it is suggested that the expression level of meiotic cohesion subunits, REC8 and SMC1B, also decrease with maternal age [161]. Correlated manifestation of increased distance in sister kinetochores is found to be evident in oocytes retrieved from old women [157], which exactly resembles alternation in oocytes from aged mice, implying that the loss of cohesion in sister chromatids might be a common mechanism to explain aneuploidies in mammals. Another loss of cohesion is elucidated to exist between homologous chromosome that synapsis in meiosis I to form bivalents and separate in anaphase I. Both in aged mice and women, there is a dramatic increase in the possibility to find univalent that is caused by premature segregation of bivalent [157, 162]. The existence of univalent pushes the oocyte to face a challenge of correct segregation, as, in this case, sister kinetochores of univalent lose the tension from homologous chromosome and tend to be attached to bipolar spindles to achieve balanced tension, which contributes to pre-division of sister chromatids [163]. The pre-division of sister chromatids will result in balanced and unbalanced segregation, but both are unexpected in meiotic process and might become a store up trouble that leads to aneuploidy [149, 162]. The mechanism that drives bivalent to univalent is still waiting to be discovered. However, one thing to be sure is that the cohesion loss within homologous chromosomes and between sister chromatids, together, lead to age-related aneuploidy, and considering to inevitable spindle disability as well as a lax supervision of meiotic check point on the error-prone process, it is unsurprised to witness aneuploidy frequently in aged oocyte.

## Genetic characteristics of ovarian aging in women

About ovarian aging, the visible disorder of ovary itself has been studied for quite a long time. Recently, researchers have endeavored to explore the mechanism of ovarian aging in depth, and achieved to find abundant new molecular and gene loci that play a part in reproductive senescence. Moreover, apart from physiological reproductive senescence, we have also paid attention to genes that might be of causal relationship with POI (primary ovarian insufficiency) or POF (primary ovarian failure), which is the pathological ovarian status caused by excessive oocyte loss, as an extreme situation of accelerated ovarian aging.

### Genome-wide association study

Without a prior expectation, Genome-wide association studies (GWAS) examine variants (single-nucleotide polymorphisms (SNPs)) in the entire genome to search SNPs with a higher frequency in cases than in normal population, and ultimately in order to find variants related to certain traits. Since menopause has long been regarded as a sign of the end of reproductive lifespan as well as a symbol of the milestone of ovarian aging, the timing of menopause is ideal to be chosen as a reproductive trait that reflects ovarian aging process. And evidence from family and twin study shows the heritability for age at menopause is 48% to 73% [164, 165], which guarantees the timing of menopause to become research highlights in genetic field of female reproductive senescence.

Nearly from 10 years ago, large GWAS study associated with the age at menopause has been carried out continuously. In 2009, two independent GWAS study first published their research results, and together they explained < 1.5% the phenotypic variation of the timing of menopause [166, 167]. Between them, Stolk et al. locks 6 significant SNPs among 3 chromosomes [13, 19, 20], involving gene *BRSK1*, *LOC284417*, *or SUV420H2* for the chromosome 19 SNPs, or *ARHGEF7* for the chromosome 13 SNP, or of *MCM8* for the chromosome 20 SNP [167]. While 13 significant SNPs distributed in chromosome 5, 6, 19, and 20 were listed by He et al. in another study, referring to the same genes in chromosome 19 and 20 and other genes like *UIMC1*, *HK3* for the chromosome 5 and *SYCP2L* for the chromosome 6 [166]. Afterwards, in 2012, Stolk et al. finished a meta-analysis of 22 GWAS studies to confirm previous 4 loci (in chromosome 5, 6, 19, 20 with loci in chromosome 13 not being confirmed) and found newly 13 loci, which pushes the boundary of the explanation strength of phenotypic variation to 2.5–4.1%. Nevertheless, genes identified in the 17 loci have been grouped into DNA repair and replication (*EXO1*, *HELQ*, *UIMC1*, *FAM175A*, *FANCI*, *TLK1*, *POLG* and *PRIM1*), immune function (*IL11*, *NLRP11* and *PRRC2A* (also known as *BAT2*)), and hormonal regulation (*FSHB*, *STAR* and *BCAR4*) [168]. Ceaselessly, using a bivariate GWAS method to reexamine the GWAS data of Stolk te.al, Perry et al. added a new variant in gene *MSH6*, functioning in DNA repairment, which affirms again the importance of DNA repair and replication in ovarian aging process [169]. However, these previous findings can explain little compared to the over 50% heritability of age at menopause, probably because of a limited case number in studied groups. Then in 2015, a study involving nearly 70,000 women conducted by Day et al. incorporates both common and low-frequency variants renovate the associated genomic loci number from 18 to 44 [170]. Not only all of the previous 18 genomic loci are reconfirmed, but also newly found genomic loci link the ovarian aging with breast cancer. More detailly, though it is not surprising to find the significant variants and associated pathways are largely related to DNA repairment, many different mechanisms are involved, including BRCA1-mediated DNA repair, mismatch repair (*MSH5* and *MSH6*) and base-excision repair (*APEX1* and *PARP2*). Moreover, 4 hits in this GWAS are supposed to near or in known POI gene (*MCM8*, *EIF2B4*, *POLG*, *MSH5* and *TDRD3*). Among those, *MCM8*, found from the first GWAS study, encodes the member of the highly conserved mini-chromosome maintenance proteins and as demonstrated by Lee et al., MCM8-9 complex is indispensable for HR which is a pathway mediating the repairment of DSBs, as has been discussed in the previous section and could lead to a genetically determined POF syndrome if it is impaired [171]. With similar function in HR, perturbation of gene *MSH5* could also lead to non-syndromic POI [172]. Later, as a supplement to a lack of East Asia ancestry, Shi te.al conducted a meta-analysis of GWAS involving 16,395 Chinese and Korean population and found a novel locus for chromosome 22 which is a genetic variant of *SFI1* encoding protein associated with spindle assembly [173]. Last but not the least, the latest and the largest GWAS study involving nearly 200,000 women of European ancestry expands the number of related genomic loci fivefold to 290, with a pathbreaking found of 6 signals at X chromosome, which not only refers to much more genetic variants related to DDR, but also highlight the potential application of gene *CHEK2* [174]. According to previous research, expression of *CHEK2*/*Chk2* (human/murine) works as a response to the signal of DNA damage (mainly DSBs) by being an inactive monomer in normal cell while activated by phosphorylation of ATM when DSBs appear and further, regulates DNA repair, cell cycle and apoptosis by phosphorylates downstream proteins [175]. In the study mentioned above, researchers delete the *Chk2* gene in female mice, and find an increase in both AMH and follicular response to gonadotrophin stimulation suggesting a lower speed of depletion in ovarian reserve which means the deletion of *CHEK2* gene is possible to defer the process of ovarian aging.

To summarize, GWAS study by now has projected at least 290 genomic loci associated with common genetic variation of ovarian aging, cumulatively explaining 31- 38% of the overall genotype-array estimated heritability, which is close to the heritability predicted by family and twin study (48–73%). The genes close to or contain significant SNPs refers to hormonal regulation, immune function and, the most important, DNA repair, which apparently needs to be further studied. Though with its own limitation that GWAS study is not capable of guaranteeing the causal association of genetic hits and the target trait, it leaves a large blank space for further research of each possible gene to ensure the association and enhance the understanding of pathways related to ovarian aging as well as finding the most significant ones. In fact, several research results have revealed some clues. For example, by now study about the reported gene co-regulation has highlighted MCM8 at the central position of the network [174]. Moreover, the genetic study of the timing of menopause is not only for in depth research of ovarian aging but also to find a better predictive biomarker in genetic aspects. Since women might gradually lose their fertility 10 years before menopause, the predict of the timing of menopause is badly in need. Compared to genetic markers, biological markers like hormones are not early enough to alarm women who is willing to pregnant at a later age and thus it is more important to carry out studies like lager scale GWAS to find common gene variants that might influence age at menopause. Lastly, the findings from GWAS studies will become the cornerstone of another well-developed and wildly-used kind of study, the Mendelian randomization studies, which regard genetic variants as instrumental variables to investigate the effects of modifiable risk factors for disease [176]. In this case, more clear causal relationship between certain traits or behaviors and age at menopause will be uncovered, providing suggestions for women to maintain fertility as long as possible. This type of study will not be discussed in this review, and readers interested are encouraged to read references [176, 177]. However, it should be noted that studies existed usually focusing on how age at menopause affects certain traits, instead of discussing how some traits impact age at menopause [178, 179]. Apparently, the later one is what clinicians most care about in order to withstand ovarian aging and is needed to be developed.

### Genetics of primary ovarian insufficiency/failure

The definition of POI and POF are nearly the same or just of slight difference, which are both characterized by menstrual disturbances (such as amenorrhea before 40), elevated FSH level and, in most cases, a depletion of follicle or oocyte [180, 181]. In this case, it might represent an excessive depletion than normal ovarian aging process. The reason why we explore genetic studies of POI/POF here is that there are more next generation sequencing (NGS) studies focusing on this specific disease with explicit diagnose criteria which makes the causal relationship between genes and certain disease more identified than GWAS study.

**Fig. 3:**
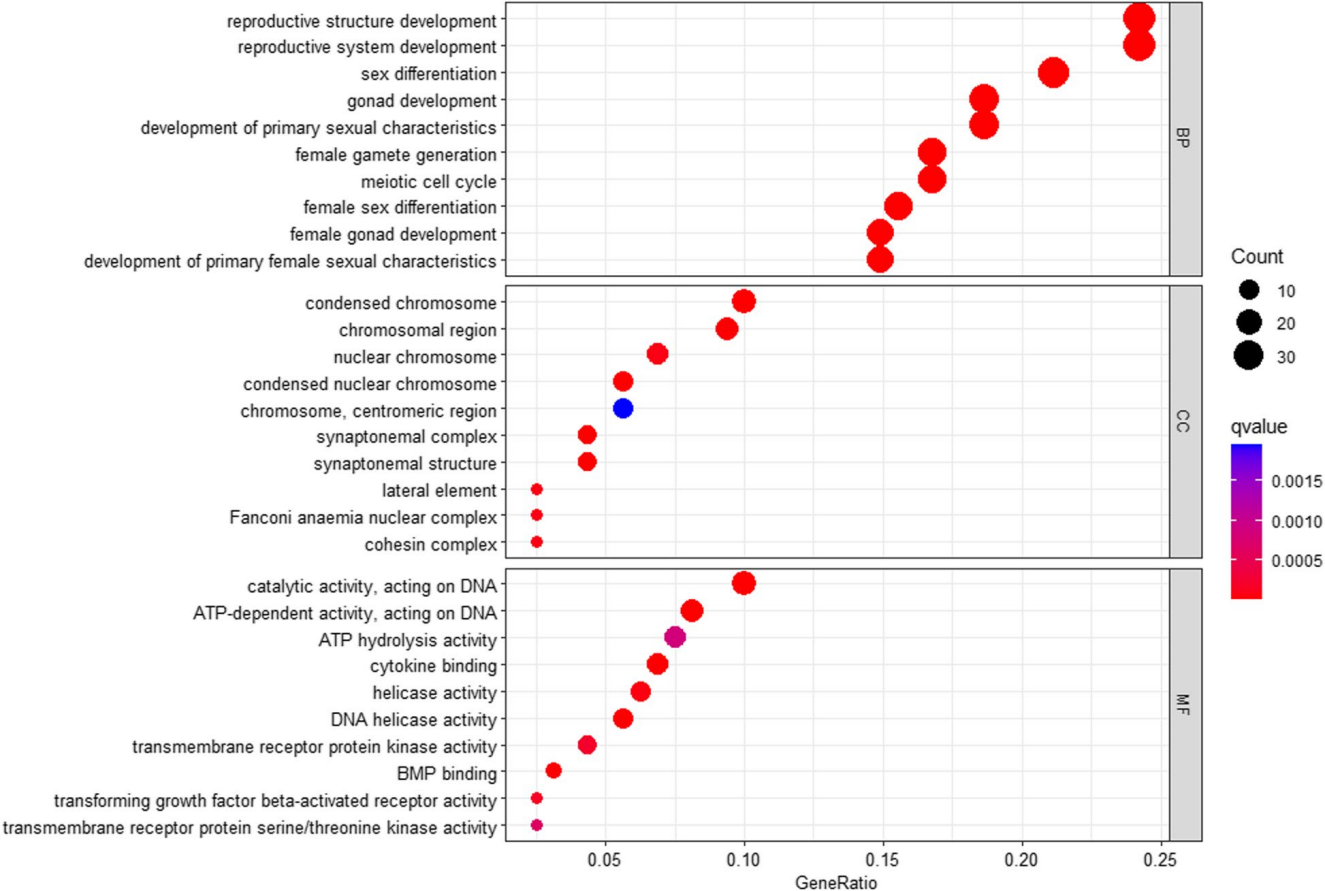
GO enrichment analysis of 168 candidate genes identified by WES in POI patients

Traditional candidate gene approach has achieved to identify a certain number of candidate genes, with part of them being functionally validated, which forms the cornerstone of POI genetics. Candidate genes found in this way include transcription factors (*FIGLA*, *NOBOX*, *NR5A1*, *WT1*, and *FOXL2*), transforming growth factor (*BMP15* and *GDF9*) and hormone receptors (*FSHR* and *AMHR2*), as summarized in review [182]. However, this approach has been proceeding slowly [182]. Over the last few decades, more and more NGS studies, especially WES (whole-exome sequencing) studies, have been carried out on POI patients both in POI pedigree and sporadic POI, expanding gene lists of POI. In POI pedigree, specific consanguineous family with POI patients are analyzed by NGS. In this way, occasionally, a certain genetic mutation could be detected. A number of new variants of genes such as *FANCM* [183], *MSH5* [184], *MCM8* [185], *FIGLA* [186], *PSMC3IP* [187], *STAG3* [188], *EIF4ENIF1* [189], and so on, have been reported and these studies, at the same time, have provided evidence for genetic heterogeneity of POI. Nevertheless, analysis of family-based large cohort has also thrived. For instance, a study done by Jolly et al. identified variants in known disease genes (*NOBOX*, *MCM8*, *PSMC3IP*, *TG*, *C3*, *GALT* and *PADI6*) and proposed candidate genes related to POI (*IGSF10*, *MND1*, *MRPS22* and *SOHLH1*) by exome-sequencing 36 unrelated families with POI [190]. Except for recruiting POI pedigree, another prevailing research method is to perform WES on candidate genes in sporadic POI patients as well as in normal control group in order to find gene variants in large population. Studies that adopt this method usually recruit much more POI patients than family-based one and are able to identify more gene variants in a single study, though the causal relationship is not so identified as family-based one. Here we have summarized 168 candidate genes identified in this way, listed in Supplementary file 1, by reviewing 19 related research [191–209]. Various genes associated with follicular development and ovulation process have been identified, whose coding products include membrane receptors, transcription factors, enzymes, structural proteins and so on. Another significant category of identified genes, as have been emphasized in GWAS studies of age at menopause, includes plenty of genes functioning in DNA damage repair (*FANCM*, *PARP1*, *PGBD3*, *FANCA*, *EXO1*, *ATM*, *MCM8*, *MCM9*, *MRE11A*, *RAD51*, *SPIDR*, *SPO11*, *RAD54L*, *RAD50*, *RAD51C*, *MSH6*, *ERCC2*, *ERCC6*), which have covered double-strands breaking repair pathway, mismatch repair pathway and nucleotide excision repair pathway. These DDR-related are also assumed to play an important role in both mitotic and meiotic process within ovary. Here we performed GO (Gene Ontology) enrichment by R (version 4.2.1) and using clusterProfiler package to better the understanding of biological significance of these genes (see Fig. 3). Not surprisingly, in biological process (BP) related pathways, enriched are female reproduction associated process, and in molecular function (MF) and cellular component (CC) analysis, genes are significantly enriched in meiotic and mitotic function, which again indicate the importance of DNA repairment. Last but not least, though plenty of candidate genes have been found and there is no doubt that more and more genes will be identified with the application of high-throughput sequencing, further research on the correlation of genotype and phenotype needs to be illuminated since only a few of them are validated by animal models and synergistical effects of candidate genes instead of effect of each single gene are supposed to be studied to better the understanding of POI etiology.

### Epigenetic study

Besides for the DNA sequence itself, other stable epigenetic changes that influence ovarian aging process exist which might include DNA modification, gene silencing and the impact of various non-coding RNA that could partly explan variability of heritability in twins’ study of age at menopause [164].

DNA methylation play a vital role in epigenetic modification on DNA. The molecular worked as DNA methyltransferase include DNMT1, DNMT3A and DNMT3B in mammals, with DNMT1 maintaining all the methylation in the genome and DNMT3A as well as DNMT3B working during embryogenesis and germ cell development [210]. Study has found an elevated expression level of *Dnmt3A* and *Dnmt3B* in older rats which might contribute to methylation of autophagy genes *Atg5* and *Lc3B*, leading to a decreased autophagy ability in the ovary of rats and somehow relates to ovarian aging [140]. Nonetheless, DNA methylation has also been researched in human female reproductive diseases. For example, epigenome wide association study designed for PCOS (polycystic ovarian syndrome) group identified 106 different methylated loci with 88 related to genes in granulosa lutein cells compared with the normal group and the study also shows a homogeneity of DNA methylation among sub-phenotypes of PCOS group [211]. Similarly, study of diminished ovarian reserve, characterized by reduced pool of primordial follicles after birth or an accelerated rate of the atretic breakdown with normal oocyte quality, also demonstrates a more abundant epimutations in mural granulosa cells, with a greater viability of DNA methylation and longer DNA methylation telomere length estimator [212], which is used as a biomarker to reflect cell replication as well as age-related pathologies [213]. In cases closed to normal population, young women (< 38 years old) with a poor ovarian response are said to manifest an accelerated predicted age when estimated by Horvath’s ‘epigenetic clock’ in white blood cells [214]. The Horvath’s ‘epigenetic clock’ is used to predict chronologic age based on DNA methylation in multi-tissue, and an accelerate age prediction might be a warning of risk during aging process [215]. Besides for DNA methylation, some modifications on histone are also supposed to influence aging process in oocytes, including acetylation, methylation and ubiquitination (reviewed by Li et al.) [216]. There is a lack of DNA and histone modification study in normal female population undergoing natural menopause, though clues from many pathophysiological groups have associated the ovarian aging process with DNA and histone modification. If proven, it might inspire more novel methods in postponing reproductive aging process since the DNA methylation and other DNA modification is possible to be reversed compared to DNA itself.

Another significant epigenetic filed is occupied by non-coding RNAs (ncRNA), which include lncRNA, miRNA, circRNA and so on. Among those, miRNA has been made an intensive study in reproductive aging. It is a short RNA with approximately 19–25 in length and acting on complementary mRNA sequence to achieve gene silencing by translational repression or mRNA decay [217]. As a matter of fact, various clues have connected miRNA with the whole reproductive process. As an instance, a review done by Donadeu et al. demonstrates the importance of miR-224, miR-378 and miR-383 in controlling the expression of aromatase during follicle development [218]. And by comparing the miRNA profiles of bovine growth and atresia follicles, researchers have found a upregulation of bta-miR-144, bta-miR-202, bta-miR-451, bta-miR-652, and bta-miR-873 in large healthy follicles than small follicles and revealed their functional role in follicular atresia [219]. Moreover miR-155 that is upregulated in the granulosa cells of PCOS is said to influence cumulus expansion, oocyte maturation and blastocyst formation in mice by targeting at gene *Smad2*, *Bcl2*, *Mecp2* and *Jarid2* [220]. And the let-7 family as well as miR-10 family has also been further studied [221, 222]. Research associated with the link between age and ncRNAs transcriptome found an upregulation of the precursor form of miRNA-1260a and miR-4262 [223]. However, though abundant findings have conformed the importance of miRNA during ovarian aging process, they come from various mammal species including pig [224], mouse [225], sheep [226] and cow [227] which means a need for further evaluation of the research results on human body. Moreover, since most of the studies done on human are focused on the influence of kinds of miRNAs on ovarian pathologies like PCOS [228], POF [227] or ovarian cancer [229], to exam whether these miRNAs play a role in natural ovarian aging process is also badly in need. At the same time, the material obtained from ovarian tissue and used in study also varies a lot from oocyte to follicular fluid, granular cell and so on, which contributes to a variation of miRNA profile and worth the attention of researchers. Here we reviewed some miRNAs existing in follicular fluid of women that might take part in the diminish of oocyte quality and oocyte pool with advanced age (see Table 1). And besides for miRNA, another ncRNA, the circRNA, forms a special covalently closed loop structures free of 5’ caps and 3’ tails and it has recently been proven to act as a sponge to absorb miRNA by complementary base paring to inhibit functioning of miRNA and regulate gene expression and protein translation [230, 231]. Study examining the ovarian circRNA respectively from old group and young group by RNA sequencing found changed expression of 401 circRNAs, of which circDDX10 is suggested to form a pathway of circDDX10-miR-1301-3p/miR-4660-SIRT3 [232], with mitochondria-localized protein SIRT3 being a family of NAD+-dependent histone deacetylase and functioning in progress of aging and cancer by interacting closely with cellular tumor antigen p53 (P53) [233]. And study focused on human granulosa cells also found an age-related variation of circRNAs among IVF patients [234]. Lastly, longer than 200 nucleotides, the lncRNA is believed to associate with ovarian pathologies and ovarian aging process, too. For example, research has reported the lncRNA HCP5 might regulate the expression of *MSH5*, which has been mentioned above as a mismatch repair, and ultimately contributes to POI to some extent [235]. To summarize, though modest success has been achieved, the variation of ncRNA is much greater than DNA itself and might act at multi-targets which make it difficult to further study on the role of ncRNA in ovarian aging process.

**Table 1:** Summary of hsa-miRNAs in follicular fluid of women that might be associated with oocyte quantity and quality

hsa-miRNAs	Main Findings	References
hsa-miR-21–5p	Differentially expressed in young and old women	[236]
hsa-miR-134		
hsa-miR-190b		
hsa-miR-99b-3p		
hsa-miR-320	Influencing embryo quality	[237]
hsa-miR-424	Differentially expressed in young and old women	[238]
hsa-miR-451	Differentially expressed referring to oocyte developmental status	
hsa-miR-27b		
hsa-miR-563		
hsa-miR-520d-3p		
hsa-miR-572		
hsa-miR-940		
hsa-miR-720		
hsa-miR-630		
hsa-miR-339-3p		
hsa-miR-483-3p		
hsa-miR-139-3p		
hsa-miR-29b		
hsa-miR-663		
hsa-miR-30c-1		
hsa-miR-198		
hsa-miR-574-5p		
hsa-miR-15a-5p	Representing poor ovary response	[239]
hsa-miR-92a	Differentially expressed referring to fertilization status	[240]
hsa-miR-130b		
hsa-miR-214	Differentially expressed referring to day 3 embryo quality	
hsa-miR-454		
hsa-miR-888		
hsa-miR-663b	Influencing blastocyst formation	[241]
hsa-miR-16-5p	Differentially expressed in young and old women	[242]
hsa-miR-214-3p		
hsa-miR-449a		
hsa-miR-125b		
hsa-miR-155-5p		
hsa-miR-372		
hsa-miR-10a-5p	Influencing oocyte development by regulating BNDF expression and representing poor embryo quality on day 3 and day 5	[243]
hsa-miR-103a-3p		
hsa-miR-1246	Maintaining oocyte quality	[244]
hsa-miR-548ae-5p		
hsa-miR-505-3p		
hsa-miR-548t-3p		
hsa-miR-513c-5p		
hsa-miR-548au-5p		
hsa-miR-320e		
hsa-miR-548au-3p		
hsa-miR-1303		
hsa-miR-484	Contributing to diminished ovarian reserve by regulating granulosa cell function through YAP1-mediated mitochondrial function and apoptosis	[245]

## Conclusion

Ovarian aging process is generally characterized by a diminished quality and quantity of oocyte or follicular pool. In order to coupe with infertility and other problems caused by such a natural process, more details are to be investigated. Principally, it is necessary to figure out whether the original trigger of this process is from brain and passes through HPO axis, or is from accumulation of harmful alternation within ovary itself. However, even just within the ovary, it needs further clarification about what is predominant among multiple factors, which include but are not limited to age-related alternation of telomere, mitochondria, biomacromolecule, oxidative stress, aneuploidy, apoptosis and autophagy. To dig into the mechanisms of ovarian aging, it is meaningful to carry out related genetic and epigenetic studies. And hopefully, recent GWAS studies have find numerous possible gene locis that might inspire researchers and more convincing studies done on POI patients by NGS have also identified plenty of gene variants. At the same time, lots of epigenetic factors including miRNA, lncRNA, circRNA, and DNA methylation have been well studied. Although a thorough understanding of ovarian aging is still waited to be established, it is promising to attempt to work out some therapeutics in an effort to delaying or even reversing this process according to knowledge in existence. And the final purpose for studying ovarian aging is to prolong reproductive life span and improve health condition of women as aging. In this case, there is still a long way to go.

## Supplementary Information

**Additional file 1.** Genes identified by WES in sporadic POI. Gene list and gene function of 169 identified genes that were found by WES in sporadic POI.

## Data Availability

Not applicable.

## Abbreviations

GWAS: Genome-wide association studies
NGS: Next generation sequencing
POI: Primary ovarian insufficiency
IVF: In-vitro fertilization
HP(O): Hypothalamic–pituitary(–ovarian)
GABA: Gamma-aminobutyric acid
KNDy: Kisspeptin/Neurokinin B (NKB)/Dynorphin (DYN)
TERT: Telomerase reverse transcriptase
TERC: Telomerase RNA component
TL: Telomerase length
Q-FISH: Fluorescence in situ hybridization method
ROS: Reactive oxygen species
EGFR: Epidermal Growth Factor Receptor
MAPK: Mitogen-activated Protein Kinase
AGEs: Advanced glycation end‐products
NF-κB: The transcription factor nuclear factor
VGEF: Endothelial growth factor
DBSs: Double-strand breaks
SSBs: Single-strand breaks
HR: Homologous recombination
MRE11: Double-strand break repair protein MRE11
RAD50: DNA repair protein RAD50
NBN: Nibrin
ATM: Serine-protein kinase ATM
H2AX: A histone protein
SAC: Spindle assembly checkpoint
UPR: Unfolded protein responses
CLPP: Caseinolytic peptidase P
BMPR1B: Bone morphogenetic protein receptor type-1B
REC8: Meiotic recombination protein REC8 homolog
mTOR: Mammalian target of rapamycin
SMC1B: Structural maintenance of chromosomes protein 1B
STAG3: Cohesion subunit SA-3
SNPs: Single-nucleotide polymorphisms
POF: Primary ovarian failure
WES: Whole-exome sequencing
BP: Biological process
MF: Molecular function
CC: Cellular component
DNMT1, DNMT3A, DNMT3B: DNA (cytosine-5)-methyltransferase 1, 3A, 3B
PCOS: Polycystic ovary syndrome
SIRT3: NAD-dependent protein deacetylase sirtuin-3
P53: Cellular tumor antigen p53
ncRNA: Non-coding RNAs
GO: Gene Ontology
